# Supraorbital Basosquamous Carcinoma Treated with Cemiplimab Followed by Sonidegib: A Case Report and Review of the Literature

**DOI:** 10.3390/biomedicines11112903

**Published:** 2023-10-26

**Authors:** Ilaria Proietti, Luca Filippi, Ersilia Tolino, Nicoletta Bernardini, Francesca Svara, Federica Trovato, Claudio Di Cristofano, Vincenzo Petrozza, Oreste Bagni, Andrea Vizzaccaro, Nevena Skroza, Concetta Potenza

**Affiliations:** 1 Dermatology Unit “Daniele Innocenzi”, “A. Fiorini” Hospital, Via Firenze, 1, 04019 Terracina, Italy; ersiliatolino@gmail.com (E.T.); nicoletta.bernardini@uniroma1.it (N.B.); francescasvara@gmail.com (F.S.); federica.trovato@uniroma1.it (F.T.); andrea.vizzaccaro@uniroma1.it (A.V.); nevena.skroza@uniroma1.it (N.S.); concetta.potenza@uniroma1.it (C.P.); 2 Nuclear Medicine Unit, Department of Oncohaematology, Fondazione PTV Policlinico Tor Vergata University Hospitale, Viale Oxfors 81, 00133 Rome, Italy; luca.filippi@uniroma1.it; 3 Pathology Unit, Department of Medical-Surgical Sciences and Biotechnologies, ICOT Hospital, Sapienza University of Rome, 04100 Latina, Italy; claudio.dicristofano@uniroma1.it (C.D.C.); vincenzo.petrozza@uniroma1.it (V.P.); 4 Department of Nuclear Medicine, “Santa Maria Goretti” Hospital, Via Antonio Canova, 04100 Latina, Italy; oreste.bagni@uniroma1.it

**Keywords:** *Basal cell carcinoma*, basosquamous carcinoma, *Squamous Cell Carcinoma*, immunotherapy, Cemiplimab, hedgehog inhibitors, Sonidegib

## Abstract

*Basal cell carcinoma* (BCC) is a skin cancer with low local aggressiveness and a low tendency to metastasize. *Basosquamous Carcinoma* (BSC) represents an aggressive histological subtype of BCC with intermediate features between *Squamous Cell Carcinoma* (SCC) and BCC. Cemiplimab is currently approved as first-line therapy in SCC and second-line therapy in BCC patients who have progressed on or are intolerant of a Hedgehog pathway Inhibitor (HHI). Our study describes the case of a 59-year-old man with BSC who was successfully treated with 5 cycles of Cemiplimab as first-line therapy and Sonidegib as second-line therapy. Currently, the efficacy of Cemiplimab against BSC and other histopathological subtypes of BCC has not been fully elucidated, as has the role of sequential or combination therapy with Cemiplimab and HHI in the management of BSC. The aim of this case report is to highlight the need to outline the use of checkpoint inhibitors in BCCs and focus attention on the synergistic role of Cemiplimab and HHIs in such a controversial entity as BSC.

## 1. Introduction

### 1.1. Basal Cell Carcinoma

*Basal Cell Carcinoma* (BCC) represents the most common tumor (approximately 15% of all neoplasms) [1,2]. New cases are not consistently recorded. The worldwide incidence is steadily increasing but cannot be estimated. BCC has a preference for people over 50 years of age and is found more frequently in men than in women [3]. It occurs in chronically sun-exposed areas, has a predominantly local malignancy, and rarely metastasizes (0.05–0.1% of cases) [4,5]. The risk factors in BCC development are: duration and intensity of ultraviolet (UV-A and UV-B) exposure, particularly in early childhood and adolescence; Fitzpatrick’s phototype; immunosuppression; and genetic predisposition [5]. At least 26 clinicopathologic variants are reported in the literature, including nodular, micronodular, superficial, morpheaform, infiltrating, and fibroepithelial (also known as Pinkus fibroepithelioma) [6]. Nodular BCC is the most common subtype, presenting as a smooth, pearly papule or nodule topped by overlying telangiectasias. Ulceration and pigmentation may occur [7]. BCC arises from basal keratinocytes and is histologically characterized by uniform and multifocal aggregates of basaloid cells that infiltrate the dermis and/or subcutis, frequently in connection with the epidermis. Histopathological variants of BCC are classified into low and high malignancy variants based on the risk of recurrence. The low-malignancy variants include nodular, superficial, fibroepithelial, and infundibulo-cystic. The high malignancy variants include micronodular, morpheaform, or sclerosing, infiltrative variants, and finally *Basosquamous Carcinoma* (BSC) [6]. At the molecular level, BCC is mainly characterized by upregulation of the Hedgehog (HH) pathway: *PTCH1* (73%), *SMO* (20%), and *SUFU* (8%) [8,9]. HH signaling assumes a key role in tissue differentiation, proliferation, homeostasis, and repair under physiological conditions. Other mutations frequently found in BCCs involve the *P53* gene (about 50%), and mutations in the *CDKN2A* locus have also been detected [5]. Treatment of BCC is usually surgical, although some forms of BCC are eligible for medical treatment or radiation therapy. A small percentage develop advanced BCC, for which systemic therapy is indicated. Hedgehog Pathway Inhibitors (HHI), Vismodegib and Sonidegib, are the only two systemic drugs approved as first-line treatments for locally advanced BCC, while Vismodegib is the only HHI approved for metastatic BCC [10,11,12]. These two HHIs have similar efficacy profiles but differing pharmacokinetic characteristics, such as half-life and volume of distribution, which result in Sonidegib exhibiting a broader distribution within the skin in comparison to Vismodegib and better tolerability [13]. However, patients develop intolerance to therapy or disease progression [1,14]. In this setting, immunotherapy plays a primary role in the treatment of most cutaneous malignancies, including BCC [15,16]. Cemiplimab is a human monoclonal antibody belonging to the Immunoglobulin G4 (IgG4) class that binds to the Programmed Cell Death Protein 1 (PD-1) receptor by blocking its interaction with PD-L1 and PD-L2. PD-1 is expressed on activated T, Natural Killer (NK), and B lymphocytes; its activation results in the maintenance of immune tolerance through inhibition of innate and adaptive responses. However, PD-1 is widely expressed on tumor-specific T cells, and its effect results in the uncontrolled proliferation of malignant cells. Diverse cellular populations express PDL-1 and PDL-2 on their surfaces, including antigen-presenting cells, B and T cells, and macrophages. These molecules can also be highly expressed by tumor cells and other cellular populations within the tumor microenvironment. Cemiplimab potentiates T-cell responses, including antitumor responses, through blocking PD-1 binding to PD-L1 and PD-L2 ligands [17,18,19]. In 2021, Cemiplimab was approved by the Food and Drug Administration (FDA), the European Medicines Agency (EMA), and the Italian Medicines Agency (AIFA) as a second-line therapy in locally advanced or metastatic BCC patients who have progressed on or are intolerant of HHI [17].

### 1.2. Squamous Cell Carcinoma

*Cutaneous Squamous Cell Carcinoma* (CSCC) is the second most common cancer in humans, right after BCC. Precise cumulative incidence is challenging to establish, but it has documented an upward trend in cSCC cases spanning several decades [20]. It originates from the uncontrolled growth of keratinocytes in the epidermis and adnexal structures; risk factors include advancing age, male gender, exposure to ultraviolet radiation (UVR), infection with β-human papillomaviruses (HPV), smoking, genetic predisposition, and immunosuppression [21]. CSCC most commonly occurs in sun-exposed areas of the skin but can be seen anywhere on the body. It often appears as a hyperkeratotic solid cutaneous lesion, often painful and ulcerated, variable in size and color. Dermoscopy evaluation may reveal characteristic features such as ulcerations, scales, telangiectasia, and linear structures radiating from the lesion [22]. Diagnosis is primarily based on an initial clinical visual examination, but confirmation typically requires a cutaneous biopsy. The majority of cSCC cases display relatively benign clinical behavior and can be successfully treated through surgical interventions or radiotherapy. However, this tumor can exhibit a more aggressive behavior, with the potential for rapid growth, invasion of surrounding tissues, and metastasis, rendering them no longer responsive to surgical or radiation interventions. Alternative treatment modalities such as chemotherapy, targeted therapy with EGFR inhibitors, and anti-PD-1 antibodies may be considered [2,23]. In 2018, Cemiplimab was approved by the FDA as a first-line treatment for patients with metastatic or locally advanced SCCs who were not candidates for surgery or curative radiation. The EMA and AIFA have since approved Cemiplimab for the same clinical indications [17].

### 1.3. Basosquamous Carcinoma

BSC accounts for approximately 1% of nonmelanoma skin cancers and frequently occurs in male patients older than 70 years [24]. Pathogenesis is controversial; the origin of BSCs has not yet been fully elucidated, but a high frequency of mutations in the HH pathway has been observed [25]. There is extensive discussion within the medical literature regarding the histological classification of BSC and Metatypical Basal Cell Carcinoma (MBCC) and whether these malignancies are identical or represent distinct subtypes of tumors. Some authors argue that BSC should be considered a distinct entity from BCC as it is characterized by the coexistence of BCC and SCC components, often grown side by side without a clear demarcation. In contrast, MBCC may exhibit squamous characteristics, but it primarily should be considered a variant of BCC [26]. Conversely, other authors argue that these two entities exhibit no substantial distinguishing characteristics to classify them as distinct BCC subtypes [27]. The recent literature considers BSC as a subtype of BCC with peculiar genetic alterations, leading to partial squamous differentiation [2,28]. This evidence could explain its aggressive behavior due to a greater tendency for recurrence (45%) and metastasis (5–10%) after treatment [29]. On clinical evaluation, BSC has non-specific features that make differential diagnosis with other keratinizing tumors extremely difficult [30]. The dermoscopic features show similarities with both cSCC and BCC: arborizing vessels, white structureless areas, keratinous masses with blood spots, ulcerations or blood crusts, white structures, and blue-gray spots [31]. Biopsy and histologic examination remain the gold standard for the diagnosis of BSC. While specific immunohistological markers have not been identified, Ber-EP4, an anti-HEA antibody that shows strong positivity in BCCs, and Epithelial Membrane Antigen (EMA), known to stain positively in cSCC, have proven to be valuable in differential diagnosis [32]. Similar to BCC, surgery is the primary treatment choice for BSC. Basosquamous lesions, while less common, are often satisfactorily treated by excision in the following conditions: early detection, limited involvement, accessible location, and clear surgical margins. Even though the local recurrence rate for BSC after surgery can reach as high as 45% [33]. For advanced or metastatic BSC, radiation and systemic therapies may be necessary [18,34]. In this setting, few cases of BSC successfully treated targeting the basaloid component with Vismodegib and Sonidegib are reported in the literature [35,36,37]. Toffoli et al. described their experience regarding Sonidegib therapy in locally advanced BSC. The first reported case involved a 59-year-old patient with a large ulceration lesion in the chest region. The histopathology report confirmed the presence of an invasive BCC with regions displaying squamous differentiation. Surgery and radiotherapy were excluded, and the patient started therapy with Sonidegib 200 mg/day. After 4 months of treatment, a regression of more than 50% of the lesion was achieved. The second presented case was a large ulcerated lesion arising in the left shoulder of an 83-year-old woman. The histopathology report revealed a sclerodermiform BCC with squamous metaplasia. Surgery and radiotherapy were deemed impractical, and Sonidegib 200 mg/day was started. After three months of therapy, a reduction in tumor size exceeding 50% was noted, and a nearly complete response was achieved within 6 months. The results observed in our patients offer initial indications that Sonidegib could potentially be efficacious in the treatment of locally advanced BSC [37].

## 2. Detailed Case Description

**Figure 1:**
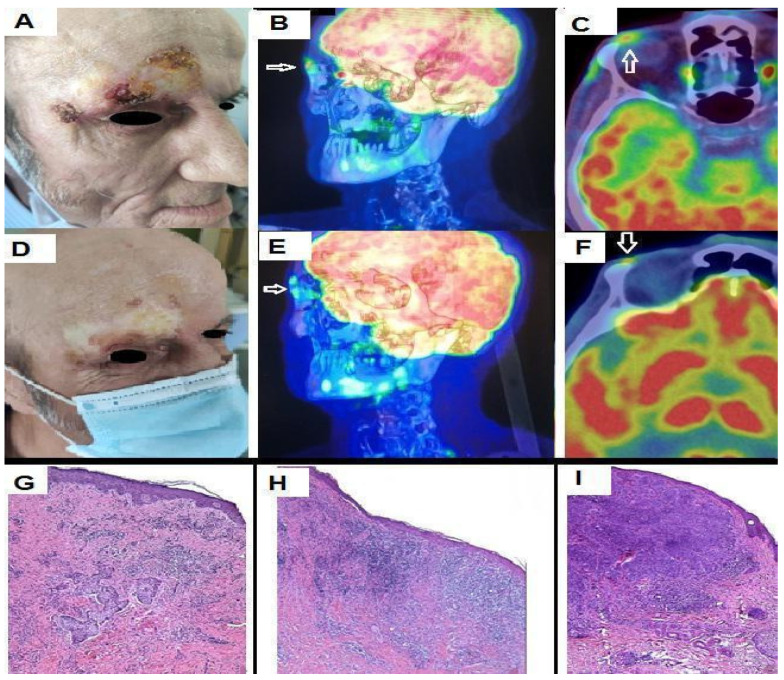
At the right fronto-orbital level, we observe an irregularly shaped, broad plaque with several shallow crusts (**A**). As indicated by arrows, PET/CT with 18F-FDG shows focal increased tracer uptake corresponding to the supraorbital lesion at presentation (**B**,**C**). After five therapeutic cycles, the patient exhibits significant clinical improvement (**D**). Also, PET/CT demonstrates a significant reduction in tracer incorporation (**E**,**F**). Section of an incisional biopsy from a large skin lesion showing an invasive cSCC before adjuvant treatment with Cemiplimab (**G**). A section of skin from the same area after medical treatment was negative for residual SCC and diffuse dermal inflammatory infiltrate (**H**). Residual BCC (**I**).

**Figure 2:**
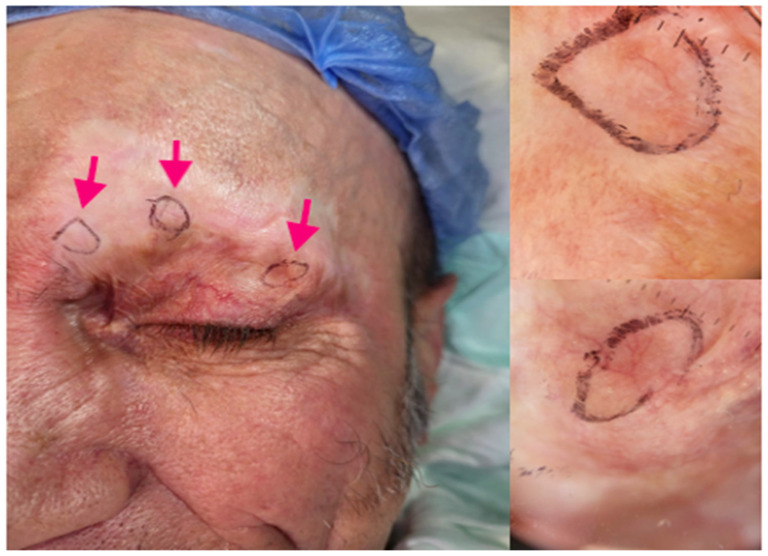
After 5 cycles of Cemiplimab, some papular lesions with dermoscopic features of BCC are clinically evident (please refer to arrows).

**Figure 3:**
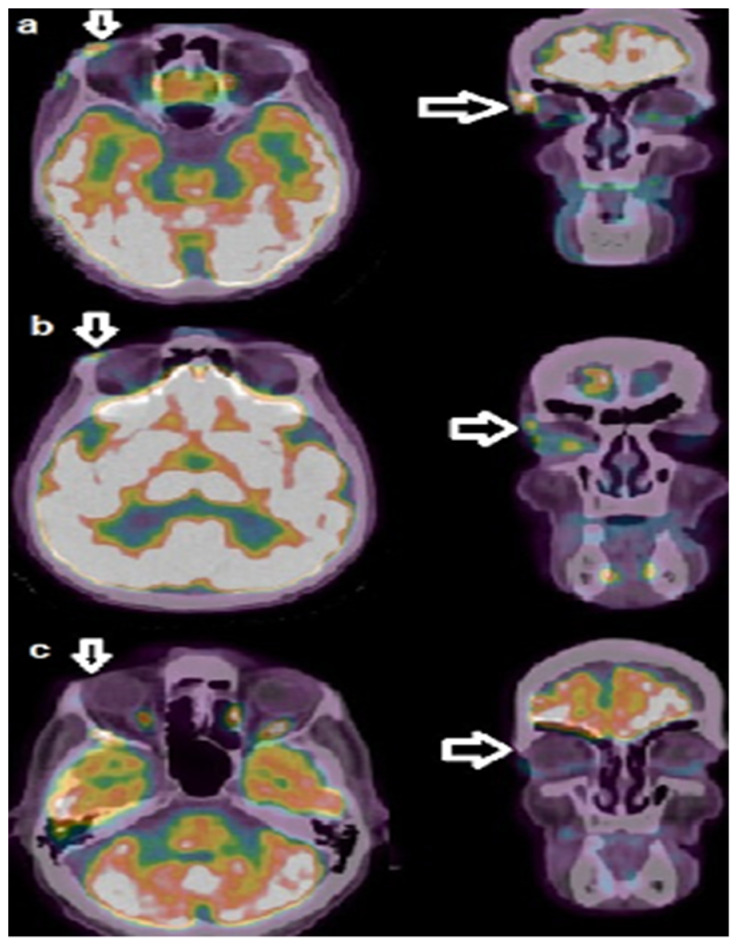
(**a**) Baseline FDG PET showed a focal area of tracer increased incorporation in the soft tissues adjacent to the supraorbital bone; (**b**) FDG PET carried at 3 months after Cemiplimab identified a small area of residual tracer uptake in the same region, indicative of a partial metabolic response; (**c**) FDG PET after 3 years of immunotherapy depicted a complete metabolic response (please refer to arrows).

We present the case of a 59-year-old man who referred to our department in March 2020, complaining of a crusty lesion in the right fronto-orbital region. The clinical evaluation showed an irregular-shaped 5 × 3 cm wide plaque with multiple superficial crusts (Figure 1A). His previous medical history was negative for relevant diseases. Laboratory exams did not show alterations. Histologic examination showed lobules and detached aggregations of eosinophilic keratinocytes with pleomorphic nuclei and some prominent nucleoli infiltrating the reticular dermis, with/without perineural and/or vascular invasion; a diagnosis of invasive SCC was taken (Figure 1G). The patient initiated treatment with an anti-PD-1 immune checkpoint inhibitor (Cemiplimab, 350 mg every 3 weeks) on 24 April 2020. Before the start of immunotherapy, he was submitted to positron emission computed tomography (PET/CT) with 18F-fluorodeoxyglucose (18F-FDG) that showed focal increased tracer uptake corresponding to the supraorbital lesion (Figure 1B,C), with a maximum Standardized Uptake Value (SUVmax) of 6.9 and a Metabolic Tumor Volume (MTV) of 2 cubic centimeters (cc). After five therapeutic cycles, he showed a remarkable clinical improvement, except for some papular lesions with the dermoscopic features of BCC (Figure 1D and Figure 2). A follow-up PET/CT, performed 12 weeks after the start of immunotherapy, demonstrated a significant reduction of tracer incorporation (i.e., SUVmax 4.8 and MTV 0.5 cc) within the supraorbital lesion, consistent with Partial Metabolic Response (PMR) according to PET criteria for evaluation of response in solid tumors, in accordance with the clinical situation (Figure 1E,F and Figure 3b). A revision of the first histologic exam highlighted the presence of basaloid cells through BerEP4 and Bcl-2 immunohistochemistry. After having clearly demonstrated the coexistence of both the squamous and basaloid components, the neoplasm was better defined as BSC. The presence of basaloid islands and significant regression of the squamous cell component were confirmed by incisional biopsy (Figure 1H,I). Considering the clinical and histological evidence of unresponsiveness of the basaloid component, Cemiplimab was interrupted after five therapeutic cycles, and Sonidegib 200 mg orally daily was started. After 3 years, PET/CT showed a complete metabolic response (Figure 3c), so the therapy was dismissed. To date, the patient is in total remission.

## 3. Discussion

This case report showed how a lesion initially described as cSCC could hide a basaloid component, leading to a wrong diagnosis. In fact, the features of BSC observed at dermoscopy are not specific and don’t provide a definitive diagnosis. Common features can be observed in cSCC and BSC, like irregular or dotted vessels within the lesion, areas of scales or crusts, and erythema [30,31]. Even histology (the gold standard for the diagnosis of BSC) can be misleading: both cSCC and BSC exhibit areas of squamous differentiation and have a tendency to invade dermis and subcutaneous tissue, causing disruption of normal tissue architecture. Both tumors typically show increased mitotic activity [38,39]. Initial diagnosis of locally advanced cSCC justifies Cemiplimab as first-line therapy. Michael R. Migden et al. evaluated, in a phase 2 study, the efficacy and safety of Cemiplimab in 193 patients with locally advanced and metastatic cSCC not eligible for surgery. Enrolled patients were treated with Cemiplimab 3 mg/kg every 2 weeks or a fixed dose of 350 mg every 3 weeks. Objective Response Rate (ORR) of 41.1% with a Disease Control Rate (DCR) of 57.1%. Cemiplimab was generally well-tolerated, with adverse events typically manageable [18,33,34]. Efficacy of Cemiplimab in BCC has been investigated in a few clinical trials. Alexander J. Stratigos et al. evaluated the efficacy and safety of Cemiplimab in locally advanced BCC in a multicenter, nonrandomized study of 84 patients who had previously received HHI therapy. Enrolled patients discontinued previous therapy due to progression, development of toxicity, or failure to achieve a better than SD response (stable disease) after 9 months of HHI therapy. Cemiplimab resulted in an ORR of 31%, with a 25% Partial Response (PR) and a 6% Complete Response (CR). Total responses were durable, with 79% of maintenance lasting beyond 6 months. Response to therapy according to BCC histotype was not evaluated in the study. Gino Kim In et al. conducted a multicenter, retrospective study of 29 patients with locally advanced or metastatic BCC who had received at least one cycle of PD-1 inhibition in any treatment line. PD-1 inhibitors used included Pembrolizumab (58.6%), Cemiplimab (31.0%), and Nivolumab (10.3%). The study revealed 17.2% of PR (5 patients) and 13.8% of CR (4 patients); the ORR was 31.0%. The percentage of those who remained in PR or CR one year after starting therapy was 100%. Three of eight patients with the basosquamous variant obtained a response (2 CR and 1 PR); among the four with the morpheaform variant, there was only one response. Both patients with micronodular variants did not respond. The study showed a trend between aggressive histology subtypes and response to therapy, with the basosquamous variant having the greatest response to anti-PD-1 [40]. In our case, after 5 months of therapy, we observed a total regression of the squamous cell component, while basaloid islands did not respond to therapy. According to these data, Cemiplimab seems to act more effectively in cSCC than in BCC. The squamous cell component of BSC could justify the greater aggressiveness of this subtype as well as the good response of this tumor to anti-PD-1; basal cell component may require a more targeted therapy to be eradicated. Hedgehog signaling pathway inhibitors are currently the only two systemic drugs approved as first-line therapy in BCC [10,11,12]. Reinhard Dummer et al., in the phase II BOLT trial, evaluated the long-term efficacy and safety of Sonidegib in patients with advanced BCC who were not candidates for surgery or radiation therapy. The study involved 230 patients who were randomly assigned in a 1:2 ratio to either receive a daily dose of 200 mg or 800 mg of Sonidegib. The study showed an ORR of 60.6% in locally advanced BCC patients for the 200 mg. Patients with metastatic BCC achieved an ORR of 8% for the 200 mg [41]. Direct comparisons of the efficacy between Sonidegib and Vismodegib in BCC treatment are limited, but their effectiveness appears to be similar, except that Sonidegib appears to exhibit greater lipophilicity compared to Vismodegib and boasts a volumetric distribution exceeding 9000 L, signifying extensive tissue distribution. Consequently, the concentration of Sonidegib in the skin is six times higher than in plasma. In theory, these findings indicate that Sonidegib undergoes more extensive distribution within the skin relative to Vismodegib. This disparity may potentially account for differences in their efficacy and toxicity profiles [42]. To date, no studies have been conducted evaluating the efficacy of HHI in BSC, alone or in combination with Cemiplimab, and few cases of therapeutic success with Vismodegib have been reported in the literature [36,37]. Toffoli et al. described their experience with Sonidegib in locally advanced BSC. They presented two cases of patients affected by BSC; both of them were not eligible for surgery or radiotherapy. Sonidegib 200 mg/day was started, and after four and three months of therapy, respectively, a regression of more than 50% of the lesion was achieved [37]. In this setting, Sonidegib could be a valid therapeutic option for the management of BSC. Elena Colombo et al. presented the first clinical results on the simultaneous and sequential full-dose administration of Sonidegib and Cemiplimab in two patients with unresectable, concurrent locally advanced BCC/SCC. In all reported cases, a complete response from both lesions was obtained, suggesting the potential synergistic action of these two therapeutic strategies [43]. Despite the common sensitivity to PD-1 inhibition, BCC and sSCC have different molecular alterations, evolution, and heterogeneous responses to immunotherapy, so they should be treated as two distinct entities [44,45].

## 4. Conclusions

We presented the first reported case of a patient affected by BSC who was successfully treated with Cemiplimab, followed by Sonidegib. Our case report is consistent with the literature: in large series, BSC is predominantly located in the head and neck area, with a higher occurrence in males and an average age distribution typically in the seventh decade of life [17]. Cemiplimab led to an important regression of the squamocellular component of the tumor, while the basal cell component was not modified. The basal cell component may have been less responsive because it was not previously treated with HHI, as happened in most clinical trials conducted on second-line Cemiplimab [1,27]. Sonidegib determined complete regression of the basal cell component, suggesting the chance of synergic activity of anti-PD-1 and HHI in the management of BSC. Our experience brings to light the need to better delineate the role of checkpoint inhibitors in the context of BCC therapy. In the treatment of a controversial entity such as BSC, there is a need to explore the broad scope of immunotherapeutics and their potential synergistic action with HHIs. We also found a close correlation between PET/CT evidence and histology, suggesting the potential benefits of metabolic imaging to monitor response to systemic treatment in non-melanoma skin cancer (NMSC). 

## Data Availability

The datasets created and analyzed during this study are available from the corresponding author upon reasonable request.

## Abbreviations

BCC—*Basal cell carcinoma*; BSC—*Basosquamous Carcinoma*; SCC—*Squamous Cell Carcinoma*; HH—Hedgehog; HHI—Hedgehog Inhibitors; IgG4—Immunoglobulin G4; PD-1—Programmed Cell Death Protein 1; NK—Natural Killer; FDA—Food and Drug Administration; EMA—European Medicines Agency; AIFA—Italian Medicines Agency; EMA—Epithelial Membrane Antigen; PET/TC—Positron Emission computed Tomography; 18F-FDG—18F-Fluorodeoxyglucose; SUV—Standardized Uptake Value; MTV—Metabolic Tumor Volume; PMR—Partial Metabolic Response; ORR—Objective Response Rate; DCR—Disease Control Rate; PR—Partial Response; CR—Complete Response; NMSC—non-melanoma skin cancer.

## References

[1] Stratigos A.J., Sekulic A., Peris K., Bechter O., Prey S., Kaatz M., Lewis K.D., Basset-Seguin N., Chang A.L.S., Dalle S. (2021). Cemiplimab in locally advanced basal cell carcinoma after hedgehog inhibitor therapy: An open-label, multi-centre, single-arm, phase 2 trial. Lancet Oncol..

[2] Guidelines CUTANEUS TUMORS NOT MELANOMA Basal Cell Carcinoma; AIOM 2021. https://www.iss.it/documents/20126/8403839/LG-AIOM-313.

[3] Basset-Seguin N., Herms F. (2020). Update in the Management of Basal Cell Carcinoma. Acta Derm. Venereol..

[4] McDaniel B., Badri T., Steele R.B. (2023). Basal Cell Carcinoma. StatPearls.

[5] Dika E., Scarfì F., Ferracin M., Broseghini E., Marcelli E., Bortolani B., Campione E., Riefolo M., Ricci C., Lambertini M. (2020). Basal Cell Carcinoma: A Comprehensive Review. Int. J. Mol. Sci..

[6] Bolognia J., Schaffer J., Cerroni L. (2017). Dermatology.

[7] Nasr I., McGrath E., Harwood C., Botting J., Buckley P., Budny P., Fairbrother P., Fife K., Gupta G., Hashme M. (2021). British Association of Dermatologists guidelines for the management of adults with basal cell carcinoma 2021*. Br. J. Dermatol..

[8] Di Nardo L., Pellegrini C., Di Stefani A., Ricci F., Fossati B., Del Regno L., Carbone C., Piro G., Corbo V., Delfino P. (2021). Molecular alterations in basal cell carcinoma subtypes. Sci. Rep..

[9] Kang S., Amagai M., Bruckner A.L., Enk A.H., Margolis D.J., McMichael A.J., Orringer J.S. (2019). Fitzpatrick’s Dermatology.

[10] Peris K., Fargnoli M.C., Garbe C., Kaufmann R., Bastholt L., Seguin N.B., Bataille V., Marmol V.D., Dummer R., Harwood C.A. (2019). Diagnosis and treatment of basal cell carcinoma: European consensus-based interdisciplinary guidelines. Eur. J. Cancer.

[11] Svoboda S.A. (2022). Systemic Targeted Treatments for Basal Cell Carcinoma. Cutis.

[12] Gutzmer R., Robert C., Loquai C., Schadendorf D., Squittieri N., Arntz R., Martelli S., Dummer R. (2021). Assessment of various efficacy outcomes using ERIVANCE-like criteria in patients with locally advanced basal cell carcinoma receiving sonidegib: Results from a preplanned sensitivity analysis. BMC Cancer.

[13] Dummer R., Ascierto P., Basset-Seguin N., Dréno B., Garbe C., Gutzmer R., Hauschild A., Krattinger R., Lear J., Malvehy J. (2020). Sonidegib and vismodegib in the treatment of patients with locally advanced basal cell carcinoma: A joint expert opinion. J. Eur. Acad. Dermatol. Venereol..

[14] Davis C.M., Lewis K.D. (2022). Brief overview: Cemiplimab for the treatment of advanced basal cell carcinoma: PD-1 strikes again. Ther. Adv. Med. Oncol..

[15] Fisher M.S., Kripke M.L. (1982). Suppressor T lymphocytes control the development of primary skin cancers in ultraviolet-irradiated mice. Science.

[16] DePry J.L., Reed K.B., Cook-Norris R.H., Brewer J.D. (2011). Iatrogenic immunosuppression and cutaneous malignancy. Clin. Dermatol..

[17] Goodman D. (2022). Cemiplimab and Cutaneous Squamous Cell Carcinoma: From Bench to Bedside. JPRAS Open.

[18] Migden M.R., Khushalani N.I., Chang A.L.S., Lewis K.D., Schmults C.D., Hernandez-Aya L., Meier F., Schadendorf D., Guminski A., Hauschild A. (2020). Cemiplimab in locally advanced cutaneous squamous cell carcinoma: Results from an open-label, phase 2, single-arm trial. Lancet Oncol..

[19] Han Y., Liu D., Li L. (2020). PD-1/PD-L1 pathway: Current researches in cancer. Am. J. Cancer Res..

[20] Leiter U., Keim U., Eigentler T., Katalinic A., Holleczek B., Martus P., Garbe C. (2017). Incidence, Mortality, and Trends of Nonmelanoma Skin Cancer in Germany. J. Investig. Dermatol..

[21] de Jong E., Lammerts M., Genders R., Bavinck J.B. (2021). Update of advanced cutaneous squamous cell carcinoma. J. Eur. Acad. Dermatol. Venereol..

[22] Kato J., Horimoto K., Sato S., Minowa T., Uhara H. (2019). Dermoscopy of Melanoma and Non-melanoma Skin Cancers. Front. Med..

[23] Corchado-Cobos R., García-Sancha N., González-Sarmiento R., Pérez-Losada J., Cañueto J. (2020). Cutaneous Squamous Cell Carcinoma: From Biology to Therapy. Int. J. Mol. Sci..

[24] Petreanu C.A., Șerban E.-D., Constantin M.-M., Savu C., Zariosu A.V., Deleanu O.C., Bucur S., Constantin T. (2021). Basal cell carcinoma-not always the ‘good guy’: Case report of a life-threatening basosquamous carcinoma and review of the literature. Exp. Ther. Med..

[25] Fotiadou C., Apalla Z., Lazaridou E. (2021). Basosquamous Carcinoma: A Commentary. Cancers.

[26] Linskey K.R., Gimbel D.C., Zukerberg L.R., Duncan L.M., Sadow P.M., Nazarian R.M. (2013). BerEp4, Cytokeratin 14, and Cytokeratin 17 Immunohistochemical Staining Aid in Differentiation of Basaloid Squamous Cell Carcinoma from Basal Cell Carcinoma With Squamous Metaplasia. Arch. Pathol. Lab. Med..

[27] Allen K.J., Cappel M.A., Killian J.M., Brewer J.D. (2014). Basosquamous carcinoma and metatypical basal cell carcinoma: A review of treatment with Mohs micrographic surgery. Int. J. Dermatol..

[28] LeBoit P.E., International Agency for Research on Cancer, World Health Organization, International Academy of Pathology, European Organization for Research on Treatment of Cancer, Universitätsspital Zürich (2006). Pathology and Genetics of Skin Tumours.

[29] Shukla S., Khachemoune A. (2020). Reappraising basosquamous carcinoma: A summary of histologic features, diagnosis, and treatment. Arch. Dermatol. Res..

[30] Gualdi G., Soglia S., Fusano M., Monari P., Giuliani F., Porreca A., Nicola M., Calzavara-Pinton P., Amerio P. (2021). Characterization of Basosquamous Cell Carcinoma: A Distinct Type of Keratinizing Tumour. Acta Derm. Venereol..

[31] Ciążyńska M., Sławińska M., Kamińska-Winciorek G., Lange D., Lewandowski B., Reich A., Pabianek M., Szczepaniak K., Hankiewicz A., Ułańska M. (2020). Clinical and epidemiological analysis of basosquamous carcinoma: Results of the multicenter study. Sci. Rep..

[32] Burston J., Clay R.D. (1959). The problems of histological diagnosis in baso-squamous cell carcinoma of the skin. J. Clin. Pathol..

[33] Migden M.R., Rischin D., Schmults C.D., Guminski A., Hauschild A., Lewis K.D., Chung C.H., Hernandez-Aya L.F., Lim A.M., Chang A.L.S. (2018). PD-1 Blockade with Cemiplimab in Advanced Cutaneous Squamous-Cell Carcinoma. N. Engl. J. Med..

[34] Ascierto P.A., Schadendorf D. (2022). Update in the treatment of non-melanoma skin cancers: The use of PD-1 inhibitors in basal cell carcinoma and cutaneous squamous-cell carcinoma. J. Immunother. Cancer.

[35] Gambichler T., Stricker I., Neid M., Tannapfel A., Susok L. (2022). Impressive response to four cemiplimab cycles of a sonidegib-resistant giant basosquamous carcinoma of the midface. J. Eur. Acad. Dermatol. Venereol..

[36] Sahuquillo-Torralba A., Llavador-Ros M., Caballero-Daroqui J., Botella-Estrada R. (2019). Complete response of a locally advanced basosquamous carcinoma with vismodegib treatment. Indian J. Dermatol. Venereol. Leprol..

[37] Toffoli L., Agozzino M., di Meo N., Zalaudek I., Conforti C. (2022). Locally advanced basosquamous carcinoma: Our experience with sonidegib. Dermatol. Ther..

[38] Yan W., Wistuba I.I., Emmert-Buck M.R., Erickson H.S. (2011). Squamous Cell Carcinoma—Similarities and Differences among Anatomical Sites. Am. J. Cancer Res..

[39] Cappilli S., Cinotti E., Lenoir C., Tognetti L., Perez-Anker J., Rubegni P., Puig S., Malvehy J., Perrot J., del Marmol V. (2022). Line-field confocal optical coherence tomography of basosquamous carcinoma: A case series with histopathological correlation. J. Eur. Acad. Dermatol. Venereol..

[40] In G.K., Nallagangula A., Choi J.S., Tachiki L., Blackburn M.J., Capone S., Bollin K.B., Reuben D.Y., Shirai K., Zhang-Nunes S. (2022). Clinical activity of PD-1 inhibition in the treatment of locally advanced or metastatic basal cell carcinoma. J. Immunother. Cancer.

[41] Sekulic A., Migden M.R., Basset-Seguin N., Garbe C., Gesierich A., Lao C.D., Miller C., Mortier L., Murrell D.F., Hamid O. (2019). Long-term safety and efficacy of vismodegib in patients with advanced basal cell carcinoma: Final update of the pivotal ERIVANCE BCC study. BMC Cancer.

[42] Dummer R., Guminksi A., Gutzmer R., Lear J., Lewis K., Chang A., Combemale P., Dirix L., Kaatz M., Kudchadkar R. (2020). Long-term efficacy and safety of sonidegib in patients with advanced basal cell carcinoma: 42-month analysis of the phase II randomized, double-blind BOLT study. Br. J. Dermatol..

[43] Colombo E., Gurizzan C., Ottini A., Caspani F., Bergamini C., Locati L.D., Marchiselli C., Alberti A., Lorini L., Licitra L.F. (2023). The association of cemiplimab plus sonidegib for synchronous cutaneous squamous cell carcinoma and basal cell carcinoma of the head and neck: Two case reports. Front. Oncol..

[44] Cives M., Mannavola F., Lospalluti L., Sergi M.C., Cazzato G., Filoni E., Cavallo F., Giudice G., Stucci L.S., Porta C. (2020). Non-Melanoma Skin Cancers: Biological and Clinical Features. Int. J. Mol. Sci..

[45] Hall E.T., Fernandez-Lopez E., Silk A.W., Dummer R., Bhatia S. (2020). Immunologic Characteristics of Nonmelanoma Skin Cancers: Implications for Immunotherapy. Am. Soc. Clin. Oncol. Educ. Book.

